# Masters of Healing: Cocaine and the Ideal of the Victorian Medical Man

**DOI:** 10.1080/13555502.2015.1124798

**Published:** 2016-01-06

**Authors:** Douglas Small

**Affiliations:** ^a^ University of Glasgow

**Keywords:** cocaine, L. T. Meade, Stories from the Diary of a Doctor, drugs, addiction, dependency, medical practitioners, medicine, masculinity, professionalism, hypnosis

## Abstract

This article offers a new perspective on the relationship between cocaine and medical practitioners in late nineteenth- and early twentieth-century Britain. Cocaine is often understood as one of a number of potentially addictive substances to which Victorian physicians and surgeons were regularly exposed, and tempted to indulge in. However, while cocaine has frequently been associated with discourses of addiction, this article proposes that it was also widely represented as a technological triumph, and that the drug was frequently used as a symbol for the scientific and moral virtues of the medical man. The argument draws on popular journalism, medical publications, and fiction to establish the cultural context of cocaine at the *fin de siècle*. In 1884, cocaine was revealed to be the first effective local anaesthetic, and this article traces the processes by which cocaine came to be regarded as the iconic achievement of nineteenth-century therapeutic science. This aura of innovative brilliance in turn communicated itself to the medical professionals who employed cocaine in their work, so that many patients and practitioners alike depicted cocaine as a most fitting emblem for the idealized selfhood of the modern medical man. This idea also informs portrayals of the drug in fiction, and I conclude with a detailed analysis of L. T. Meade’s 1895 short story, ‘The Red Bracelet’ (published in the *Strand Magazine* as part of Meade’s series, ‘Stories from the Diary of a Doctor’), as an example of the way in which cocaine functions as metaphor for the physician’s unassailable moral primacy and technical excellence.

In 1892, the Irish addiction specialist Conolly Norman penned ‘A Note on Cocainism’ for the *Journal of Mental Science*. Norman began his article with a warning: ‘[Though] a comparatively new drug […] cocaine is more seductive than morphia; it fastens upon its victim more rapidly, and its hold is at least as tight.’ Another alarming circumstance connected with cocaine was that ‘up to the present time the largest number of its victims appear, unfortunately, to have been medical men’.^1^ Norman’s introduction gives us a glimpse into the anxieties surrounding the relationship between cocaine, addiction, and medical professionals at the *fin de siècle*.^2^ The cocaine alkaloid had only truly entered public consciousness in 1884, when it was discovered that a weak solution of the drug would, if introduced into the eye, neutralize its sensitivity to pain, whilst leaving the patient conscious. In effect, cocaine was the first practical local anaesthetic. Within a short space of time, however, reports began to proliferate of its toxicity and addictive potential. In January 1886, the *British Medical Journal* opined: ‘we have already found out that this sweet rose of our therapeutic bouquet has its bitter thorn’.^3^ Medical practitioners were thought to be particularly vulnerable to cocaine habituation, paying a ‘heavy tribute’ to addiction partly because of ‘their ready access’ to dangerous drugs and partly because they laboured under a greater burden of work and responsibility than other similarly educated gentlemen.^4^ Cocaine, the ‘bitter thorn’ concealed in the ‘therapeutic bouquet’, was an implied threat to practitioners’ personal and professional standing: an easy road by which the difficulties of work could run to degeneration.

It is important to understand, however, that cocaine existed in a deeply paradoxical situation in the late-Victorian and Edwardian periods. In discussing early cocaine use in the United States of America, Joseph F. Spillane observes that ‘evidence of early medical praise for the benefits of cocaine and the subsequent concerns over its dangers can be misread as a simple progression from the former to the latter’.^5^ In this period – both in Britain and abroad – cocaine was simultaneously understood to be a dangerous, potentially addictive substance, and a remarkable medical innovation. When its anaesthetic powers were revealed, it was widely applauded as having ‘immediately wrought a complete revolution’ in medicine, and as ‘a discovery to captivate the imagination of mankind’.^6^ The chemical was hailed as a profoundly transformative and triumphant achievement of modern medical science, and the knowledge that it could be misused did little to dispel the glamour surrounding it. Cocaine also had a similarly dualistic significance for the *fin-de-siècle* medical man. In this period it is common for cocaine to be represented as not merely a great discovery in its own right, but *the* iconic feat of nineteenth-century humanitarian scientific endeavour. This lustre in turn transferred itself to representations of the medical gentlemen who employed it. In both medical and popular depictions, the physician who could administer cocaine to banish pain or perform surgery seemed the heroic incarnation of a set of scientific and personal virtues, an ideal image of authority, self-discipline, and expertise. In contemporary sources, cocaine is allocated a totemic role in defining the *fin de siècle*’s sense of its own transcendent modernity, and that innovative brilliance is incarnated in the form of the practitioner who could bring the drug to bear for the benefit of his patients.

This article examines an alternative strand present in the discourse surrounding cocaine use and the medical professional: as well as egregious failure, cocaine could illustrate the astonishing moral and scientific successes of these men. For almost 20 years after the revelation of its anaesthetic function, cocaine was enthusiastically received by both the therapeutic and lay communities. The result of this was that the alkaloid acquired a deeply personal significance for the public image as well as the self image of medical personnel; among physicians and patients alike, the notion developed that the consummate power of the modern medical man was best represented by cocaine, the ‘consummation’ of modern therapeutic chemistry.^7^ This idea also informs portrayals of the drug in fiction, and I conclude with an analysis of L. T. Meade’s 1895 short story ‘The Red Bracelet’ as an example of the way in which cocaine functions as an almost mystical manifestation of the physician’s unassailable moral primacy, as well as his Aesculapian exceptionalism.

## I. A revolutionary technology

The anaesthetic function of cocaine had been first announced at the Congress of the Heidelberg Ophthalmological Society in September 1884, and it was in the field of optical surgery that cocaine was to have its most immediate and most enduring impact. Awake, but immune to pain, a patient could cooperate with the operating surgeon and keep them apprised of the progress of their work. Cocaine also eliminated the risk that the patient’s unconscious movement would damage the eye being operated on. Henry Power, president of the British Medical Association’s Ophthalmology Section, asserted that ‘in the discovery of cucaine [*sic.*], a new era seems to have dawned’.^8^ However, cocaine also possessed strong attractions for the medical community as a whole. The compound appeared to realize a long-standing – almost immemorial – aspiration of medicine: it could remove pain without either the danger or the stupefaction attendant upon ether and chloroform. Indeed, as we shall see, a recurrent feature of late-Victorian cocaine discourse is the implied contrast between the safety and efficiency of the new drug, and the danger and inconvenience that were widely believed to be inherent to the use of chloroform.

Chloroform had been widely used as an anaesthetic for almost 40 years by the time cocaine entered public consciousness. But its first introduction into the operating room in 1847 was followed, ‘within months’, by its first fatality.^9^ Anaesthetic death remained a consistent (though comparatively rare) danger of surgical activity until the end of the century. By the time cocaine began to be become popular, conventional anaesthesia was still, in the eyes of both doctors and patients, ‘a dangerous and unreliable practice’.^10^

There were also less obviously fatal tendencies associated with chloroform. Contemporary works on anaesthesia warned physicians that:

One of the most annoying results from [chloroform] anaesthesia is the production of erotic hallucinations, which occur during recovery from the narcosis; and, as is the rule in subjective impressions, they are very vivid. This danger, if there were none other, would be sufficient to necessitate the presence of a second medical man during the administration of an anaesthetic.^11^

The risks of scandal, or ‘annoyance’, attached to anaesthetic unconsciousness were accompanied by further concerns. Some commentators expressed the worry that, by annulling not only the patient’s pain, but also their consciousness, chloroform might annul the surgeon’s ability to see their patient as a fellow human being. Ian A. Burney describes the fear that:

the anaesthetized patient could neither through a conscious act of will nor through the corporeal language of pain resist the surgeon’s actions; the surgeon, on the other hand, might view the patient as a mere object, the surgical scene evacuated of the beneficial limitations imposed by human empathy.^12^

When compared with the threat of chemically enforced passivity and dehumanization, cocaine seemed a pleasing alternative. Under the influence of cocaine, the patient remained awake, remained aware, and – in a sense – remained human, despite the operation being performed on them. In this light, the celebratory, almost climactic, language associated with the drug becomes easier to understand: not only was the revelation of cocaine’s effects dramatically unexpected, those effects seemed almost miraculous, and the medical world responded accordingly.

In the year after the Heidelberg Ophthalmological Congress, there was a rush to identify and publish new uses for the drug. In the first six months of 1885, there were 67 pieces on cocaine in the pages of the *British Medical Journal*, proselytizing its value in ‘operations on the vagina and urethra, in dentistry, ophthalmic surgery, in vaccination, in operations on the nose and larynx, vomiting, mammary abscess, in cancer, scalds, circumcision, neuralgia, hay fever, senile gangrene, nymphomania [and] sea sickness’.^13^ To mark the end of the first year of its use, the surgical journal *Annals of Surgery* published a comprehensive survey of the drug and its applications. To the list of uses enumerated in the *British Medical Journal* it added (among others): cocaine’s efficacy as a mild antiseptic; its value to women whose nipples were sore or chapped from nursing; as a tonic for digestive troubles and against wasting syndrome; to relieve the pain of caustic injections for syphilis; as a therapy for morphine and opium addiction; and (significantly for the drug’s later career) as a stimulant and as an aphrodisiac.^14^ Improving on the 2% solution used in early experiments, it could now be administered via hypodermic injection, cocainized Vaseline, cocaine-impregnated gelatine disks, or a specially designed hydraulic spray.^15^ The article confidently concluded:

From this general retrospect the large and important place which has been established for cocaine within the year following its first public demonstration at the Heidelberg Congress is apparent.

As the sum of the whole, it may be said that, so broad is its application, and so frequently is it indicated by reason of its power to substitute ether and chloroform, and thus do away with the disadvantages and dangers of these drugs, added to the fact that the patient is continued in consciousness while pain is prevented, it occupies the position of an ideal anaesthetic more nearly than any other drug now known.^16^

Cocaine not only appeared to be the ‘ideal anaesthetic’, but held out the promise that modern science had, in the last years of the nineteenth century, brought forth a modern panacea.

The eagerness surrounding the drug also communicated itself to the popular press. In papers and magazines, cocaine inspired an almost sacerdotal enthusiasm. Headlines announced ‘The Latest Cocaine Miracle’, and the *Scotsman* declared it to be ‘one of the blessed instruments of [Christ’s] pain-removing and peace-instilling mission’.^17^ This rapturous approval was accompanied by the sense that cocaine represented the culmination of nineteenth-century technology, that this single innovation – the effortless conquest of pain – could embody a century’s worth of scientific investigation. In 1900, surgeons working at Chicago’s Cook County Hospital perfected a technique to inject cocaine into the spinal canal, thus allowing severe operations to be carried out without the use of chloroform, since the injection directly anaesthetized the central nerves in the spine. The English paper, the *Daily News*, began its report on the discovery with a rhetorical flourish: ‘When the question is asked, “What is the greatest discovery of the nineteenth century?” the reply not infrequently made is, “The suppression of pain in surgical operations”.’^18^ Only a few years before, the doctor and prolific cultural commentator Alfred J. H. Crespi had expressed his awe at the ‘meteor-like rapidity’ with which cocaine had travelled from the fringes of discovery to become ‘the prized possession of millions’, and an ‘invaluable’ weapon in ‘the armoury of the modern scientific surgeon’.^19^ For Crespi, the transformation of Brazilian or Peruvian coca leaves into cocaine, and the ever-decreasing cost of the process, mirrored the power of modern engineering to transmit electricity over ‘vast distances’ at a ‘rapidly decreasing cost’.^20^ Crespi applies the same modernizing rhetoric of distance, economics, and utility to describe both electricity and cocaine. He imbues the drug with a galvanic dynamism, so that it captures the insubstantial energies of the age in a material, medicinal form.

Nowhere was cocaine more grandly ennobled than in a piece published in the *London Reader* in 1902. Entitled ‘Progress of the Nineteenth Century’, it is a type of centennial panegyric, celebrating the many advances made over the preceding hundred years. It states:

This century received from its predecessors the horse; we bequeath the bicycle, the locomotive, and the automobile.

We received the goosequill; we bequeath the fountain pen and the typewriter.

We received the scythe; we bequeath the mowing machine.

We received the sickle; we bequeath the harvester.

We received the hand printing press; we bequeath the rotary.

We received twenty-three chemical elements; we bequeath eighty.

We received the tallow drip; we bequeath the arc electric light.

We received the sailing ship; we bequeath the steamship.

After 16 similar contrasts the final two lines conclude:

We received unalleviable pain; we bequeath asepsis, chloroform, ether, and cocaine.

We received the average duration of life of thirty years; we bequeath forty years.^21^

Throughout, ‘Progress of the Nineteenth Century’ maintains a consistent format: the impoverished technological legacy of the eighteenth century, contrasted with the wonders attained during the nineteenth, and now bestowed upon the twentieth. The piece builds through the arts, though agriculture, through physics, chemistry, and industry, until it reaches its conclusion in the medical sciences. Here, cocaine becomes, literally, the crescendo of nineteenth-century innovation. As the unfaltering march of progress climbs from the reign of George III to the death of Victoria, its final destination is the gift of 40 years of life.^22^ The verses transform a multitude of scientific discoveries into a single endeavour, with a single, profoundly humanitarian, attainment at its end. Figured like this, cocaine stands for the culmination of one hundred years of industrial and scientific revolution.

## II. The exceptional drug and the exceptional self

If cocaine, then, could be understood as the apogee of Victorian social and scientific improvement, it was not difficult to assign this same consummate quality to the medical gentleman who put the drug into service. Equipped with an ideal implement of healing, the practitioner could represent a professional and personal ideal.

This ideality can be clearly seen in Marian von Glehn’s ‘A Day in a Hospital’, published in the *Leisure Hour* in February 1890. Von Glehn’s article describes her visit to the Royal London Ophthalmic Hospital. She writes that, while speaking to one of the nurses:

A messenger interrupted asking the nurse to get her patients ready, as Mr — (the operating surgeon) would be upstairs in about ten minutes. One could not escape an involuntary shudder. And yet, such are the wonderful properties of the newly discovered cocaine, that the operating theatre is robbed of half of its terrors…

[In the lobby-room outside the operating theatre] I watched the nurses with their quiet manner inspiring their patients with courage and confidence as they dropped the magic drug into the eye which was to deaden and allay the pain. […] Thoughts must turn to prayer before such spectacles of human suffering and endurance.

Nor was the endurance displayed by the patients alone striking. To anyone unfamiliar with hospital life, it might well seem noteworthy that operations requiring the greatest nerve and technical dexterity should be performed by the same surgeons who for hours already had been giving the most concentrated attention to the out-patients’ cases below.^23^

Von Glehn equates the therapeutic effectiveness of cocaine with the capabilities of both the nurses and the surgeons. The ‘magic’ of the drug mingles with the inspiring – almost seraphic – competence and compassion of the women who apply it, so that both appear to develop organically out of each other. The rapidity and ease with which the drug takes effect, having to be but lightly ‘dropped’ into the eye, mirrors the evanescent presence of the nurses, who drift with gentle efficiency through the lobby outside the operating room, easily dispensing both cocaine and a reaffirmed confidence in the power of medical science. Significantly, the religious timbre of the writing also strongly suggests the image of the nurses as a body of female angels (or a community of *religieuses*) dedicated to the service of god-like male practitioners. These men are, in turn, inexhaustible, commanding, and encircled with a refined atmosphere of isolation and anonymity. Both groups, though fulfilling different roles, reflect an underlying superhuman excellence, which is ultimately enabled by ‘the wonderful properties’ of cocaine.

The ophthalmologists of the Royal London Hospital are remote divinities, but a more affable medical ideal can be found in the person of the heroic Dr Campbell from Harry Stillwell Edwards’s 1896 novel, *Sons and Fathers*.^24^ At one point, Campbell and his assistant Edward are called upon to perform a delicate operation to save the sight of a wife and mother of two, by removing her glaucoma. The chapter in which the surgery takes place is given the distinctly messianic title ‘The Hand of Science’.^25^ It is Campbell’s own hand that wields the cocaine and the surgical scalpel, but both he and the drug he administers take on an aspect of supernatural intervention; together, the doctor and the drug are portrayed as the avatars of an almost deified scientific majesty. When Stillwell Edwards describes Campbell entering his patient’s home, he writes: ‘The famous practitioner, a tall shapely figure, entered, and as he removed his glasses he brought sunshine into the room, with his cheery voice and confident manner.’^26^ Physically graceful, charismatic, and reassuring, Campbell metaphorically returns ‘sunshine’ to the family home, just as he will soon use cocaine and surgical skill to return light to the lady of the house in a more literal sense.

The doctor takes only the briefest interval to make his diagnosis, and so ‘deliberate’ is he, ‘in every word and action’, that ‘the occasion was already robbed of half its terrors, so potent are confidence, decision, and action’.^27^ Campbell and Edward immediately go to their business:

There was no chloroform, no lecture. With the simplicity of a child at play, the great man went to work. Turning up the eyelid, he dropped upon the cornea a little cocaine, and selecting a minute scalpel from his case, with two swift, even motions cut downward from the centre of the eye and them from the same stating point at right angles. The incisions extended no deeper than the transparent epidermis of the organ. Skilfully turning up the angle of this, he exposed a thin, white growth – a minute cloud it seemed to Edward.

“Another drop of cocaine please,” the pleasant voice of the oculist recalled him, and upon the exposed point he let fall from the dropper the liquid. Lifting the little cloud with keen pincers, the operator removed it, restored the thin epidermis to its place, touched it again with cocaine and replaced the bandage. The strain of long hours was ended; he had not been in the house thirty minutes.^28^

The entire passage not only emphasizes Campbell’s personal skill, but also creates an equivalence between his surgical incisiveness, and the efficacy of the cocaine. Campbell’s cuts are swift and precise, and the narration imitates this by taking place in rapid, succinct clauses and statements. The surgery is punctuated by efficient drops of cocaine that form the counterpoint to Campbell’s quickness by instantly annulling the pain of the operation.

The initial mention of chloroform is also significantly phrased. Not only does it imply the comparative safety of the operation (since it removes the risks associated with the older drug), it also affirms that Campbell’s presence in the house is a consoling, compassionate one. As we have seen, chloroform was connotative not only of the risks of surgery, but also of a dehumanizing distance between practitioner and patient.^29^ The absence of chloroform is tied to the absence of any unpleasant attribute in Campbell’s character; just as he does not employ the soporific chloroform, Campbell does not coldly keep his distance or delay to ‘lecture’. He operates on his patient, Mrs Montjoy, with quickness and confidence, and this assured speed of action is also the wider hallmark of his personality. In the same manner, the ‘great man’ is seen to act with ‘with the simplicity of a child at play’, an image which testifies to Campbell’s technical *sprezzatura*, while also imparting to him a humane, even domestic aspect. Cocaine and Campbell’s surgical adroitness transform a difficult operation into easy playfulness. After his work is concluded, he happily dandles the Montjoys’s youngest son on his knee, further establishing a familial intimacy between patient and practitioner.^30^ In Stillwell Edwards’s novel, cocaine permits a type of treatment which is both emotionally comforting, and technologically astounding. Thirty minutes of cocaine and professional expertise are all it takes for Campbell to restore sight to his patient, and hope to her house. The manners of the ideal practitioner evolve from the mechanism of cocaine.

Harry Stillwell Edwards and Marian von Glehn in effect combine cocaine and medical personnel into a fantasy of scientific sanctity. While theirs was a view from outside the magic circle of surgery, medical men themselves also used cocaine as a way to articulate their identities as practitioners – the drug became a symbol through which to express the professional conditions of their lives, and their emotional responses to those conditions.

Perhaps surprisingly, it was not only the elite of ophthalmic specialists who developed a personal investment in the employment of cocaine – so too did the humble General Practitioner. In discussing the drug, many of these men focused their observations on the contrast between cocaine and older varieties of anaesthesia, and on how the newer compound might promote a better relationship between them and their patients. In 1900, J. Eustace Webb, a GP in Aberdeenshire, submitted a paper to the *Scottish Medical and Surgical Journal* called ‘The Use of Hydrochlorate of Cocaine in Private Surgical Practice’. For the previous six years, Webb had used cocaine anaesthesia almost exclusively for the minor surgeries commonly required of a GP because ‘in general private practice the dread of the patient to take a general anaesthetic, and often the difficulty of getting an administrator [anaesthetist] are felt as distinct disadvantages to operative work being done’.^31^ From Webb’s point of view, cocaine not only negated the practical obstacles associated with other drugs, it was more reassuring for patients as well.

Another GP, William Semple Young, reached the same conclusions. Working in a small country practice in Garlieston in the west of Scotland, he found that ‘there are many people who dread chloroform so much, that they decline to take it, unless practically coerced into doing so’.^32^ Semple Young was so impressed with the drug that, when, in 1898, the time came for him to produce his MD thesis for the University of Glasgow, his subject was ‘Cocaine as a Local Anaesthetic’. Like J. Eustace Webb, he applauded the benefits that cocaine could offer the General Practitioner:

Cocaine has this advantage over chloroform, that no assistant is required. It is also safer, as the patient is conscious all the time, and can give timely warning in case of any untoward symptoms coming on. Its price is also (which to many patients is a serious item) very much less than that of chloroform.^33^

The fact that cocaine was cheaper to use, less dangerous, and more easily administered than other anaesthetics, made it obviously appealing to the General Practitioner. Without chloroform, the GP was no longer a potential figure of ‘dread’ to his clients.

These practical considerations were also accompanied by a more personal investment in the drug on the part of some medical professionals. Semple Young’s MD thesis contains a short but significant passage where he explains why he selected cocaine as his subject. The work begins with his reminiscences of a boyhood terribly afflicted by toothache, ‘what Burns has termed “the Hell o’ a’ diseases”’, and goes on to explain: ‘When as a student I heard such glowing reports of the use and action of cocaine in tooth extraction, I determined, that if ever I had the opportunity, I would study the action of the drug in this particular direction.’^34^ Semple Young’s initial consideration of cocaine as a dental anaesthetic expands into an assessment of how ‘invaluable’ the alkaloid is to all branches of modern medicine.^35^ Semple Young establishes at the outset that his interest in cocaine derives from the discomforts of his childhood – discomforts which medicine was then powerless to alleviate. When he hears about cocaine as a student, it is an inspiring moment for him since it illustrates how far *fin-de-siècle* medicine has come and what it is now capable of. Behind his analysis of its physical capabilities, Semple Young imbues the drug with an emotional resonance, since it signifies not only the progress of medical science but also his own progress as a doctor. There is a professional and personal intimacy inherent in the thesis’ description of the drug: cocaine not only provides a consoling connection between his childhood distress and his adult vocation, but – by making it the subject of his dissertation – cocaine is now about to make him an MD, furthering his standing once again. The introduction to Semple Young’s thesis implies a sort of professional bildungsroman, in which cocaine plays a defining part; as a practitioner, his assessment of cocaine is blended with his ideas about his life and work.

A similar presentation of the compound can be seen in ‘Recent Advances in Surgery and Medicine’, an article which appeared in the *Edinburgh Review* in October 1888. The piece unites a discussion of how cocaine has ‘revolutionized’ medicine with detailed depictions of the personality, social status, and professional situation of the medical man.^36^ The piece documents the many inconveniences that the practitioner could face in his vocation, but it also paints a picture of how cocaine – as an iconic medical technology – could combine with the practitioner’s heroic nature to help him transcend these obstacles. The opening pages state that:

[The life of the medical man] is peculiarly a life of untiring warfare; he is always in the thick of the battle with disease, and, moreover, he has to exert himself strenuously to hold his own among his brethren and to earn a livelihood. The doctor is almost the only educated man claiming to be treated and regarded as a gentleman, and often having the tastes and instincts of that class, who commonly works for and looks for payment to classes far beneath his own in the social scale. […] A medical career consequently lacks, and must always lack, those social amenities and advantages which attract able and accomplished men in such numbers to other liberal callings.^37^

This summary of the problems inherent in the late-Victorian medical profession is fairly straightforward: the medical man must constantly ‘battle’ to preserve his patient from disease; he must then battle almost as hard to get that patient to pay him; and, finally, he must struggle to retain his own sense of himself as a respectable middle-class gentleman. ‘Untiring warfare’, ‘exertion’, ‘strenuousness’, and ‘conflict’ are the defining activities of a life where *déclassé* is almost as fierce an enemy as disease. The prevalence of these difficulties can be seen from Roy Porter’s remark that, by the 1880s, ‘few [doctors] secured a competent living before they were approaching forty’.^38^

Amidst these larger anxieties there were also more specific irritations that doctors might encounter. ‘Recent Advances’ addresses the concern that, while other branches of medical practice could delight in a torrent of new devices and discoveries, the General Practitioner might feel himself depressed by the comparative plainness of his day-to-day employment:

It has been said that the advance in [traditional] medicine has not equalled that in surgery, and can hardly be placed in comparison with it. […] Medicine, moreover, has had its field greatly curtailed of late. Many obscure and obstinate diseases, which were included in its province, and for which until recently comparatively little could be done, have been transferred to the realm of the surgeon; and, while obstinately intractable as long as medicines were alone administered, are found to admit of ready and successful treatment at the hands of the surgeon. Physicians can no longer claim to be the gentlemen and scholars of the profession, and to regard surgeons as their humble dependents.^39^

This passage expresses an alarming possibility: in being left behind technologically, the physician might also be left behind socially. It suggests that the once dependable hierarchies of the medical profession could be disrupted by the alterations of the new age.

But ‘Recent Advances in Surgery and Medicine’ was quick to reassure its readers that physicians were not mere relics of the past – they too could participate in the era of technological medicine. After the passage above, there follows the remark:

Still, there have been triumphs, of which we may mention a few: the treatment of rheumatic fever with salicylate of antipyrin, the induction of sleep with chloral, paraldehyde, urethane, and hypnone, and the introduction of cocaine, the last invaluable as an internal remedy.^40^

These new drugs – particularly the ‘invaluable’ cocaine – are held out as evidence that the work of the physician has not stood still whilst other fields have leapt ahead. These chemicals are tied to the professional and social status of physicians: the drugs fortify their position against the vicissitudes of the modern world, and infuse the General Practitioner’s work with a precious quantum of technological lustre. Just as surgical specialists had had their abilities ‘immensely enlarged’ by cocaine, General Practitioners could also claim the drug as a source of pride in themselves and their work.^41^

On a wider scale, the economic and social disadvantages that practitioners suffered through could be said to pale before the medical man’s own personal virtues. The business of medicine was ‘a most useful occupation, one affording unbounded scope to the most enlightened and far-reaching mind [… and offering] the most complete and constant union of those three qualities which have the greatest charm for pure and active minds – novelty, utility and charity’.^42^ Financial concerns are also presented as being immaterial, since the work of medicine is ‘carried out without that slavery to it which greed begets and fosters’.^43^ A sense of noble, charitable endeavour is imagined to ameliorate the practitioner’s labour. This superiority also communicates itself to the mind and body:

He has scope for muscular exercise; he has always to be acquiring new information, which keeps the mental organism employed; and as he soon discovers that to make his presence endurable to the sick he must be serene and cheerful, he acquires a temper of serenity and cheerfulness.^44^

Furthermore, the medical professional enjoyed a more robust physicality than the average man. In medicine, ‘Recent Advances’ maintained, ‘the average of health, though not the duration of life, is far above the common average in other professions’.^45^ The medical gentleman’s moral and intellectual capacities were also more finely developed: ‘It is surely fair to hold that as in every search for knowledge we may strengthen our intellectual power, so in every practical employment of it we may, if we will, improve our moral nature.’^46^ More than any alternative career, in medicine a man might rely upon his work to enhance his nature. While his employment might put him beyond many of the comfortable sureties of middle-class life, the practitioner could reassure himself that he, in his person, represented the heroic ideal of middle-class professional masculinity: knowledgeable, physically fit, and morally assured.

‘Recent Advances in Surgery and Medicine’ enfolds its discussion of cocaine’s technological cachet into a larger depiction of the superior nature of the medical individual. Cocaine revolutionizes surgery, and compensates physicians for their ‘curtailed field’.^47^ But this is only a small part of a narrative in which technical innovation and individual exceptionalism combine to dispel the annoyances of medical work. Together, cocaine and the selfhood of the practitioner form two sides of the same coin, one which repays the medical man for the obstacles of his chosen career. From surgical specialists to family doctors, the drug is unified with the personality of the practitioner who uses it; cocaine is presented as the most ‘invaluable’ of modern therapeutic innovations, and the medical professional possesses the most incomparable of modern selves.^48^

## III. ‘The Red Bracelet’

The idea that the medical man constituted the apex of middle-class personal and professional existence is taken to an even greater extreme in L. T. Meade’s short story, ‘The Red Bracelet’ (published in the *Strand Magazine* in January 1895). Here, we are presented with a doctor whose morality and scientific assuredness are so potent that he can combine cocaine and surgical intervention to bestow these qualities upon another character. In Meade’s story, cocaine occupies a pivotal position, as it manifests the practitioner’s idealized personal virtues and allows them to effect his patient both physically and emotionally.

‘The Red Bracelet’ is one of a series of medical mystery tales – with the overarching title *Stories from the Diary of a Doctor* – that Elizabeth Thomasina (or ‘L. T.’) Meade produced for the *Strand* between 1893 and 1895. These narratives revolve around patients suffering from particularly mysterious or outré ailments, and the attempts of the gifted physician, Dr Clifford Halifax, to diagnose their condition. In a metatextual move, the *Stories from the Diary of a Doctor* were advertised as being a collaboration between Meade and ‘Clifford Halifax, M.D.’ himself – an act which was clearly supposed to buttress their appearance of medical authenticity. Despite the fictionality of Clifford Halifax, Meade really did obtain medical details for the stories from the Metropolitan Police Surgeon Edgar Beaumont.^49^ Throughout her career, Meade was keenly attuned to the narrative allure of new technologies and medical procedures. Janis Dawson remarks that Meade’s work was characterized by a high degree of ‘literary marketplace savvy’.^50^ Indeed, her *Stories from the Diary of a Doctor* were ‘inspired by the phenomenal success of Arthur Conan Doyle’s Sherlock Holmes series’.^51^ However, not only was Meade ‘particularly adept at following literary trends’, but early in her career she ‘learned to exploit sensational incidents and topical issues to construct best-selling [stories]’.^52^

This sense of exciting scientific contemporariness is particularly evident in the various works that Meade produced for the *Strand Magazine*. In her novel *The Brotherhood of the Seven Kings*, for example, one of the victims of the villainous Madame Koluchy is found to have mysterious star-shaped wounds on his face and neck, which are later revealed to have been caused by exposure to ‘constant powerful discharges of cathode and X-rays’.^53^ Likewise, each story was prefaced by the statement:

These Stories are written in collaboration with a medical man of large experience. Many are founded on fact, and all are within the region of practical medical science. Those stories which may convey an idea of the impossible are only a forecast of an early realisation.^54^

Meade’s medical mystery stories are, therefore, specifically written to attract readers through a combination of literary and scientific fashionability. The portrayal of cocaine in ‘The Red Bracelet’ not only illustrates its technological topicality, but also potentially harks back to the drug’s appearance in the early Sherlock Holmes stories, where it was used to convey Holmes’s rigorously intellectual and professional manner of living.^55^ In ‘The Red Bracelet’ cocaine similarly manifests the rational and moral capability of Dr Clifford Halifax.

The case at the centre of the story is partly medical, partly supernatural, and partly a domestic drama. Dr Halifax is consulted by a man named Stafford who is deeply concerned about his daughter, Molly. She suffers from congenital blindness and although Mr and Mrs Stafford have taken her to the finest oculists in Europe, nothing can be done to cure her. Molly has also recently fallen under the control of an unscrupulous man by the name of Basil Winchester. After a chance encounter, Winchester has been able to hypnotize Molly so that she longs only to be with him and is incapable of thinking of anything else. Stafford asks Halifax to counter Winchester’s control of his daughter, since her hypnotic obsession is seriously threatening her health. When Halifax arrives at the Staffords’s home in Yorkshire, it is revealed that Basil Winchester is able to project his influence into Molly’s mind through a red coral bracelet that he has given her. Though unfamiliar with hypnotic techniques, Halifax is able to use his personal aura of authority to get Molly to eat and rest a little. She remains sick, however, and Halifax theorizes that it is Molly’s blindness that makes her unusually vulnerable to Winchester’s power.

The break in the case comes when Halifax realizes that, though her vision is so severely impaired that she is effectively blind, Molly has a subliminal perception of the presence of light and darkness. He deduces that her eyes are really perfectly intact, but obscured behind a dense, fleshy layer of tissue. With the aid of cocaine, Halifax performs a rapid operation to remove the obstruction. Her sight restored, Molly is freed from her false lover’s influence and when Winchester next meets her, Molly rejects him. He is soon thereafter arrested for forgery. At the story’s end, Molly sends the red bracelet to Halifax as a gesture of thanks.

At the heart of the story is an explicitly moral message, but this moralizing is conveyed almost entirely through a series of densely interlinked metaphors and symbolic associations. Although Molly has been hypnotized by Winchester, the hypnosis does not result merely in his having a zombie-like control over her. Rather, it causes her to become emotionally fixated on him. The story uses purposefully ambiguous language to describe Winchester’s mesmeric power. He is persistently referred to as possessing an ‘influence’ over Molly, or as having created a ‘strange craze’ or ‘infatuation’ in her (pp. 546, 558, 556). Even more tellingly, her father states that: ‘Badly as [Winchester] has treated her, her overpowering passion for him is beyond all reason’ (p. 549). By describing the hypnosis in explicitly emotional terms, Meade obscures the distinction between mesmerism and a more conventional romantic obsession. Coupled with these descriptions, there are frequent references to Molly’s refusal to submit to her parents’ well-meaning instructions that she should break off her attachment to Winchester. At their first meeting, Mr Stafford tells Halifax that ‘[Molly] is deaf to our entreaties. She thinks of nothing morning, noon, or night, but this man’ (p. 547). Halifax later incredulously urges Molly to ‘think of your parents’ but she flatly responds: ‘My father and mother were opposed to our marriage, but I cared nothing for their opposition’ (p. 553). Here, Molly’s hypnosis is clearly identified as a metaphorical rendering of an unsuitable attachment. Underlying the medical mystery of ‘The Red Bracelet’ is a moral and domestic issue: a highly emotional young woman is deceived into forming a connection with an unworthy man, she abandons filial obedience for desire, and his eventual mistreatment causes her to distress and sicken herself. The hypnosis is a pseudo-scientific disguise for a consuming passion that destroys Molly Stafford’s power of rational thought, and causes her to defy social and domestic propriety.

This interpretation is reinforced by the fact that – even before she meets Winchester – Molly is characterized as emotionally disordered and vulnerable. In her ‘overpowering passion’ for him, she is alternately described as ‘laughing wildly’, ‘giving way to hysteria’, and piteously clinging to her mother like a child (p. 551). Molly’s blindness is explicitly identified as the origin of her emotional state. She mournfully recalls: ‘I never thought that love – love of this sort – could come into the life of a blind girl’ (p. 553). After examining her, Halifax gives a comprehensive analysis of her condition:

Few doctors believe in the well-known phrase ‘a broken heart’, but if anyone was likely to die of this malady, the girl over whom I was now watching would be the one. Her blindness and her particularly nervous and highly strung temperament would all conduce to this effect. […] Her illness is due to a strange and overstrained condition of the imagination. All her thoughts are turned inwards. Her blindness adds much to this condition. If only I could give her back her sight! (p. 555)

This diagnosis conjoins Molly’s blindness, her unhealthy emotional introversion, and her susceptibility to Winchester’s malign influence. Her defective sight is the underlying factor; in restoring it, Halifax also restores her emotional coherence and frees her from Winchester’s mesmeric affliction. Molly’s lack of sight is, therefore, also a lack of insight; unable to see, she is also symbolically unable to see Basil for the scoundrel he truly is. In this vein, there are numerous faintly punning references in the text to the fact that, in lacking the sense of sight, Molly also lacks common sense. Halifax calmly informs his patient that: ‘You have parted for the time being with common sense […] I mean to bring that precious possession back to you’ (p. 552). In the final pages of the story, Molly’s restored eyes are twice referred to as ‘her new possession’ (p. 560). In allowing the girl to possess the power of sight, Meade establishes that Halifax has gifted his patient with a new self-possession; in remedying a physical ailment, he has undone a social and emotional malignity.

In this context, the use of cocaine to remedy Molly’s deformed eyes is also a means by which to restore moral and domestic correctness. The symbolism in the story is constructed around cocaine’s physiological properties, since without the drug it would be impossible to accomplish the surgery that realigns Molly’s visual and social acuity. In ‘The Red Bracelet’ cocaine is an agent of both technological and moral advancement, a refinement in medical technology that effects a more refined personal conduct. Meade highlights the moral superiority of cocaine by contrasting it with the older anaesthetic chloroform. When Halifax suggests the surgery to Molly’s parents, her mother anxiously enquires:

‘Will the operation be painful? Will it be necessary for you to use chloroform?’

‘No [Halifax responds]; I shall put cocaine into the eye – don’t be alarmed, Miss Stafford will feel no pain.’ (p. 558)

Mrs Stafford’s question explicitly differentiates Halifax’s employment of cocaine from the ‘dangerous and unreliable’ threat of chloroform anaesthesia.^56^ It allows L. T. Meade to confirm the more developed condition of modern medicine which has done away with the crude instruments and practices of the past. Given the metaphorical significance of Molly’s blindness, though, the contrast between cocaine and chloroform also reiterates the story’s larger contrast between moral and immoral behaviour, between the light of social awareness and the darkness of irresponsible desire. Chloroform, which rendered the subject unconscious, and which could provoke delusional erotic fantasies,^57^ is easily relatable to the blackness surrounding Molly, both because of her defective eyes, and because of the hypnotic occlusion of her perceptions by Winchester. Cocaine, on the other hand, is connotative of both a literal and social awareness. In juxtaposing cocaine and chloroform, Meade affirms cocaine’s status as a tool of moral clarity and consciousness. In ‘The Red Bracelet’ cocaine confirms that innovations in medical technology can also be seen as improvements in moral nature.

Simultaneously, Meade also makes it clear that cocaine is a type of material synecdoche for the idealized virtues of Halifax’s own personality. In fact, before he uses cocaine to open Molly’s eyes, the main way in which he treats her is through imposing his own unflinching rationality and moral authority onto her. ‘The Red Bracelet’ establishes a clear opposition between Basil Winchester, the evil hypnotist, and Clifford Halifax, the good doctor. Just as Basil influences Molly towards selfishness and passion, the doctor influences her towards self-control and domestic deference. But where Winchester uses deception and mesmerism, Halifax uses his own personal rectitude and willpower. When Molly tells him that she is too distraught to eat, Halifax calmly counters: ‘“No, that is folly. You are giving way to a feeling of hysteria. This is causing your father and mother great unhappiness. Your throat is not closed, you only imagine it.”’ Molly obediently goes to eat, and Halifax remarks: ‘“She didn’t even attempt to struggle against my stronger will”’ (p. 551). Later, in conversation, he tells her:

‘I am going to exercise my will over yours.’

‘You have done so already,’ she answered. ‘I eat when you tell me; I sleep when you wish me to; I don’t feel wicked when I am with you. I even begin, just a little, a very little, to take an interest in my father and mother again. Basil used to make all the rest of the world a blank.’ (p. 556)

Halifax’s will has the power to restore Molly’s sense of right and wrong, and her awareness of her social obligations. As a doctor, Halifax perfectly encapsulates the middle-class, professional, and masculine virtues of self-restraint, rationality, and ethical firmness. He is so potent an ideal that, when Molly succumbs to girlish emotion and hysteria, he can transfuse his moral conviction into her, and restore her to physical and psychological coherence. For L. T. Meade the medial professional represents the supreme apotheosis of middle-class values, and in curing bodily ills, the medical man also cures the infirmities of the soul.

Cocaine, then, is really Halifax’s moralizing will condensed into material form. Through the innovative new drug, Halifax is able to excise the sclerotic membrane that veils Molly’s eyes, and purge the moral infection of hypnosis. Interestingly, Winchester has a comparable relationship with the titular red bracelet that he gives to Molly. The hypnotist is absent for the majority of the story, and he transmits his desires to the girl through the bracelet. Molly describes it as ‘a part of the man I love’ and it is ‘a link between her and him’ (pp. 553, 549). If the red bracelet manifests Basil’s wicked influence over Molly, then cocaine has a similarly talismanic connection to Dr Halifax. When the physician drops the cocaine into her eye, the drug forms a chemical connection between Molly’s weakened nature and his superior one. In ‘The Red Bracelet’ medicine is seen to be a social and moral process, as much as it is a physical one. As a doctor, Halifax becomes the superhuman realization of middle-class professional and personal virtues, and cocaine becomes the incarnation of his miraculous, curative will.

## IV. Conclusion

In March 1886, *Chambers’s Journal* published an article in praise of cocaine, ‘the new discovery which [had] agreeably startled the world of medicine towards the end of the year 1884’.^58^ It proudly announced the new drug to be the heir of chloroform and ether, and an anaesthetic that had now freed physicians, surgeons, and patients from the ‘discomforts’ and ‘inconveniences’ of those older compounds.^59^ The article’s final lines announced:

In the present prosaic condition of the world, when the surfeit of new discoveries seems to have bred in this connection the familiarity which produces the conventional contempt, it is refreshing to draw attention to a discovery which has surpassed the ordinary standard of greatness sufficiently to enable it to figure as a wonder of the age. Cocaine flashed like a meteor before the eyes of the medical world, but, unlike a meteor, its impressions have proved to be enduring; while it is destined in the future to occupy a high position in the estimation of those whom duty requires to combat the ravages of disease.^60^

Cocaine’s destiny was to be more conflicted than *Chambers’s Journal* foresaw, however. The following year, the *Illustrated Police News* had wryly responded to the first reports of cocaine poisoning by remarking: ‘cocaine is getting into discredit’.^61^ This sense of disappointment and ‘discredit’ was to persist as medical and popular writers became increasingly aware of the drug’s ‘dangerous and alluring’ charms, and of the ‘hopeless, endless chain’ of addiction that it might forge around the unwary.^62^

For many, though, cocaine was still haloed, ‘like a meteor’, with the celestial luminescence of discovery, and this same light also encircled the practitioner who made use of the drug in his daily work. Employed for healing, rather than for recreation, cocaine could signify the monumental achievements of *fin-de-siècle* medicine, and the transcendent stature of the medical man. For writers like Alfred J. H. Crespi, the neat syringe or pipette that held the chemical seemed to contain all the aggregated triumphs of a century of technological innovation. Among medical men and patients alike, the drug articulated a glamorous image of the practitioner’s superior selfhood. And for authors like L. T. Meade, the solution of cocaine became a moral solution, a dissolved form of the medical professional’s masterful ideality. In *fin-de-siècle* fiction, medical literature, magazines and journals, cocaine forms part of a beatific constellation of virtues contained within the medical gentleman.

## Funding

This work was supported by the Wellcome Trust under Grant WT108549AIA.

## Disclosure statement

No potential conflict of interest was reported by the author.

Douglas Small *University of Glasgow* douglas.small.2@glasgow.ac.uk

## Footnotes

^1^ Conolly Norman, ‘A Note on Cocainism’, *Journal of Mental Science*, 38.161 (April 1892), 195–99 (p. 195).

^2^ For a more in-depth examination of the discourses surrounding substance addiction and medical men in the late-Victorian period, see Brenda Mann Hammack, ‘Phantastica: The Chemically Inspired Intellectual in Occult Fiction’, *Mosaic: A Journal for the Interdisciplinary Study of Literature*, 37.1 (2004), 83–100; Barry Milligan, ‘Morphine-Addicted Doctors: The English Opium-Eater, and Embattled Medical Authority’, *Victorian Literature and Culture*, 33.2 (2005), 541–53; and Susan Zieger, *Inventing the Addict: Drugs, Race, and Sexuality in Nineteenth-Century British and American Literature* (Amherst: University of Massachusetts Press, 2008), pp. 127–55.

^3^ ‘Special Correspondence’, *British Medical Journal*, 1.1305 (2 January 1886), 40.

^4^ J. J. Graham Brown, ‘Notes on the Treatment of the Diseases of the Nervous System’, *Scottish Medical and Surgical Journal*, 4 (January–June 1899), 499.

^5^ Joseph F. Spillane, *Cocaine: From Medical Marvel to Modern Menace in the United States* (Baltimore: Johns Hopkins University Press, 2000), p. 25.

^6^ Simeon Snell, ‘Presidential Address on Some Points of Progress in Ophthalmic Surgery’, *British Medical Journal*, 2.1489 (13 June 1889), 58; Alfred J. H. Crespi, ‘Some Recent Scientific Advances’, *Gentleman’s Magazine*, 1906 (October 1889), 391–406 (p. 391).

^7^ ‘The Operation for Cataract’, *Newcastle Weekly Courant*, 9 June 1894, p. 5.

^8^ Henry Power, ‘An Address Delivered at the Opening of the Section of Ophthalmology and Otology’, *British Medical Journal*, 2.1283 (1 August 1885), 207.

^9^ Ian A. Burney, *Bodies of Evidence: Medicine and the Politics of the English Inquest, 1830–1926* (Baltimore: Johns Hopkins University Press, 2000), pp. 138–39.

^10^ Burney, *Bodies of Evidence*, p. 141.

^11^ George Foy, *Anaesthetics Ancient and Modern* (London: Baillière, Tindall, and Cox,1889), p. 114.

^12^ Burney, *Bodies of Evidence*, p. 145.

^13^ Virginia Berridge, *Opium and the People: Opiate Use and Policy in Nineteenth and Early Twentieth Century Britain* (London: Free Association Books, 1999), p. 221.

^14^ See James E. Pilcher, ‘Cocaine as an Anaesthetic: Its Status at the Close of the First Year of its Use’, *Annals of Surgery*, 1.3 (January 1886), 51–66 (pp. 58–61).

^15^ Pilcher, ‘Cocaine as an Anaesthetic’, p. 54.

^16^ Pilcher, ‘Cocaine as an Anaesthetic’, p. 66.

^17^ ‘Latest Cocaine Miracle’, *Hampshire Telegraph and Sussex Chronicle*, 26 December 1885, p. 12; ‘Cocaine’, *Scotsman*, 29 August 1887, p. 7.

^18^ ‘Painless Surgery’, *Daily News*, 6 October 1900, p. 5.

^19^ Crespi, ‘Some Recent Scientific Advances’, pp. 398–99.

^20^ Crespi, ‘Some Recent Scientific Advances’, p. 394.

^21^ ‘Progress of the Nineteenth Century’, *London Reader*, 25 January 1902, p. 377.

^22^ It is worth noting that the closing lines of ‘Progress of the Nineteenth Century’ reflect a general mood of optimism about substantially increasing lifespans throughout the 1800s and early 1900s. Pat Jalland writes: ‘Life expectancy at birth in England and Wales began to rise from about 40 years in 1850 to about 52 for males and 55 for females by 1911–12. The death-rate began to decline slowly from 22 per 1,000 in the 1870s to 13 per 1,000 by 1910.’ Similarly, the percentage of the British population over 65 rose from 4.7% in 1861 to 5% by 1901. See Pat Jalland, *Death in the Victorian Family* (Oxford: Oxford University Press, 1996), p. 143. Commentators consistently remarked on these changes throughout the last decades of the nineteenth century. As comparatively early as 1881, the *Spectator* wrote: ‘We all know that whatever be the truth throughout the world, the “expectation of life”, that is, the chance of longevity, has, in the well-drained and carefully-fed cities of the West, increased to a perceptible degree.’ ‘The Improvement of Human Health’, *Spectator*, 23 July 1881, p. 11.

^23^ Marian Von Glehn, ‘A Day in a Hospital’, *Leisure Hour*, February 1890, pp. 277–81 (p. 279).

^24^ Though Stillwell Edwards was an American writer, *Sons and Fathers* was serialized in the British press throughout 1896.

^25^ Harry Stillwell Edwards, ‘Sons and Fathers’, *Nottinghamshire Guardian*, 10 October 1896, p. 6.

^26^ Stillwell Edwards, ‘Sons and Fathers’, p. 6.

^27^ Stillwell Edwards, ‘Sons and Fathers’, p. 6.

^28^ Stillwell Edwards, ‘Sons and Fathers’, p. 6.

^29^ See Burney, *Bodies of Evidence*, p. 145.

^30^ Stillwell Edwards, ‘Sons and Fathers’, p. 6.

^31^ J. Eustace Webb, ‘The Use of Hydro-Chlorate of Cocaine in Private Surgical Practice’, *Scottish Medical and Surgical Journal*, 7 (July–December 1900), 46.

^32^ William Semple Young, *Cocaine as a Local Anaesthetic* (Glasgow: University of Glasgow, 1898), p. 51.

^33^ Semple Young, *Cocaine as a Local Anaesthetic*, pp. 36–7.

^34^ Semple Young, *Cocaine as a Local Anaesthetic*, pp. 3–4.

^35^ Semple Young, *Cocaine as a Local Anaesthetic*, p. 39.

^36^ ‘Recent Advances in Surgery and Medicine’, *Edinburgh Review*, 168.344 (October 1888), 491–515 (p. 506).

^37^ ‘Recent Advances in Surgery and Medicine’, pp. 491–92.

^38^ Roy Porter, *Disease, Medicine and Society in England, 1550–1860* (Basingstoke: Macmillan, 1993), p. 51.

^39^ ‘Recent Advances in Surgery and Medicine’, pp. 510–11.

^40^ ‘Recent Advances in Surgery and Medicine’, p. 511.

^41^ ‘Recent Advances in Surgery and Medicine’, p. 507.

^42^ ‘Recent Advances in Surgery and Medicine’, p. 492.

^43^ ‘Recent Advances in Surgery and Medicine’, p. 494.

^44^ ‘Recent Advances in Surgery and Medicine’, p. 494.

^45^ ‘Recent Advances in Surgery and Medicine’, p. 493.

^46^ ‘Recent Advances in Surgery and Medicine’, p. 493.

^47^ ‘Recent Advances in Surgery and Medicine’, p. 511.

^48^ For descriptions of cocaine as an ‘invaluable’ drug, see ‘Recent Advances in Surgery and Medicine’, p. 511; Crespi, ‘Some Recent Scientific Advances’, p. 399; and Semple Young, *Cocaine as a Local Anaesthetic*, p. 39.

^49^ Janis Dawson, ‘Rivaling Conan Doyle: L. T. Meade’s Medical Mysteries, New Woman Criminals, and Literary Celebrity at the Victorian Fin de Siècle’, *English Literature in Transition*, 58.1 (2015), 54–72 (p. 57).

^50^ Dawson, ‘Rivaling Conan Doyle’, p. 69.

^51^ Dawson, ‘Rivaling Conan Doyle’, p. 56.

^52^ Dawson, ‘Rivaling Conan Doyle’, p. 55.

^53^ See L. T. Meade and Robert Eustace, ‘The Brotherhood of the Seven Kings’, *Strand Magazine*, 15 (January–June 1898), 649–64 (p. 664).

^54^ L. T. Meade, ‘The Red Bracelet’, *Strand Magazine*, 9 (January–June 1895), 545–61 (p. 545). Further page references for ‘The Red Bracelet’ will be given in parentheses in the main body of the text.

^55^ Perhaps counter-intuitively for a modern audience, Arthur Conan Doyle makes it clear in the opening passages of *The Sign of Four* that Sherlock Holmes is not a cocaine addict, nor does he take cocaine for recreation. Rather, he only resorts to the drug to keep his mind ‘stimulated’ and ‘clarified’ when he has no case on hand. See Arthur Conan Doyle, *The Sign of Four*, ed. by Peter Ackroyd and Ed Glinert (London: Penguin, 2001), pp. 5–6. Instead of being a distraction from work, or a pleasurable indulgence, Holmes’s cocaine habit is in fact only a stop-gap and ‘artificial substitute’ for the mental and professional exertion of detection. For a fuller examination of Sherlock Holmes’s cocaine habit, see Douglas Small, ‘Sherlock Holmes and Cocaine: A 7% Solution for Modern Professionalism’, *English Literature in Transition*, 58.3 (2015), 341–60.

^56^ Burney, *Bodies of Evidence*, p. 141.

^57^ See Foy, *Anaesthetics Ancient and Modern*, p. 114.

^58^ ‘Cocaine’, *Chambers’s Journal*, 3.114 (6 March 1886), 145–47, (p. 145).

^59^ ‘Cocaine’, *Chambers’s Journal*, p. 145

^60^ ‘Cocaine’, *Chambers’s Journal*, p. 147.

^61^ ‘Everybody’s Column’, *Illustrated Police News*, 3 December 1887, p. 2.

^62^ Norman, ‘A Note on Cocainism’, p. 196; ‘Old and New’, *Scots Observer*, 8 February 1890, p. 334.

